# Early postoperative bone scintigraphy in the evaluation of microvascular bone grafts in head and neck reconstruction

**DOI:** 10.1186/1746-160X-3-20

**Published:** 2007-04-20

**Authors:** Jonas Schuepbach, Olivier Dassonville, Gilles Poissonnet, Francois Demard

**Affiliations:** 1 Department of Otolaryngology, Head and neck surgery, University Hospital Inselspital Berne, Freiburgstrasse 10, CH-3010 Berne, Switzerland; 2 Centre Antoine Lacassagn, 33, av.de Valombrose, F-06189 Nice, France

## Abstract

**Background:**

Bone scintigraphy was performed to monitor anastomotic patency and bone viability.

**Methods:**

In this retrospective study, bone scans were carried out during the first three postoperative days in a series of 60 patients who underwent microvascular bone grafting for reconstruction of the mandible or maxilla.

**Results:**

In our series, early bone scans detected a compromised vascular supply to the bone with high accuracy (p < 10-6) and a sensitivity that was superior to the sensitivity of clinical monitoring (92% and 75% respectively).

**Conclusion:**

When performing bone scintigraphy during the first three postoperative days, it not only helps to detect complications with high accuracy, as described in earlier studies, but it is also an additional reliable monitoring tool to decide whether or not microvascular revision surgery should be performed. Bone scans were especially useful in buried free flaps where early postoperative monitoring depended exclusively on scans.

According to our experience, we recommend bone scans as soon as possible after surgery and immediately in cases suspicious of vascularized bone graft failure.

## Background

Reconstruction of mandibular defects caused by trauma or tumour surgery has long been a major problem in maxillofacial surgery. Since advances in microsurgical techniques allow transfer of vascularized bone grafts, several pedicled osteomuscular flaps have been described. At the present time, free scapula, iliac crest and fibular grafts are most often used and have been shown to be reliable [1-4]. The successful incorporation of a bone graft depends on an adequate blood supply and vital osteoblasts. Many different methods of monitoring vascular patency and viability of bone graft have been described. Inclusion of a skin island in bone grafts allows conventional monitoring techniques including direct clinical observation, pinprick testing as well as surface temperature probes and pulsoxymetry. Despite its widespread use, monitoring of the skin flap is not always reliable in the assessment of overall viability, especially in mandibular reconstruction which often requires multiple osteotomies [5]. Color duplex sonography is reported to be a reliable and non-invasive monitoring technique [6,7] but may fail if the anastomosis is not superficial. Angiography can reveal the patency of anastomoses but cannot show microcirculation, and its invasiveness with tendency to cause spasm and thrombosis precludes routine use. Implantable venous Doppler probes first described by Swartz provide "real-time" information regarding both arterial and venous flow and seem to be a promising tool for intraoperative and postoperative monitoring [8-10]. Magnetic resonance angiography may play a role in the future. Bone scintigraphy using Technetium 99 m methylene disphosphonate (MDP) and dicarboxyproprane diphosphonate (DPD) has found widespread use in assessment of bone blood flow and metabolism, including monitoring of maxillo-facial bone grafts. It is non-invasive, simple and effective in postoperative assessement. Single photon emission computed tomography (SPECT) and 3-D reconstructions reportedly allow more precise imaging than conventional planar scanning [11]. Most authors report carrying out scintigraphy at approximately the seventh postoperative day, with the earliest reported cases 48 hours after surgery [11]. These procedures showed good correlations with clinical outcome. However, taking into account that the majority of thrombi occur within the first two postoperative days [12], we performed bone scintigraphy within the first 12 to 72 hours after surgery. The correlation of the bone scintigraphy with classical monitoring techniques was used to assess the microvascular status with regard to revision surgery of the graft anastomoses.

## Patients

Sixty patients (39 men and 21 women, aged 35 to 82 years, mean 60 years) who underwent autogenous microvascular bone grafting for reconstruction of the mandible or maxilla in the period from 1.1.1997 to 1.8.2004 were included in this retrospective study. The reason for bone grafting was malignancy in 41 patients (40 squamous cell carcinomas, 1 malignant melanoma), osteoradionecrosis in 13 patients, ameloblastoma in 4 patients and necrosis of preceding bone graft in 2 patients. All patients underwent primary reconstruction. Fifty-four grafts were transferred from the fibula and 6 from the scapula.

All fibula grafts were used for mandibular reconstruction after resection of the symphysis in 23 patients, the mandibular body in 53 patients, the ramus in 37 patients and the condylar process in 14 patients. In 9 patients, no fibular osteotomies were performed, in 31 patients one osteotomie and in 14 patients 2 osteotomies. Fifty fibular flaps were transferred with skin pedicle.

Scapular grafts were used when fibular grafts could not be harvested because of insufficient blood supply to the foot (n = 3), when reconstruction with fibular grafts had failed (n = 2) and for reconstruction after maxillectomy (n = 1). In 3 patients with scapular graft, one osteotomie and in 3 patients no osteotomie was performed. All scapular grafts were transferred with a skin pedicle.

All patients had the first scintigraphic examination within 72 hours after completion of surgery. Bone scans were performed on the day of surgery in 2 patients, on the first postoperative day in 40 patients, the second day in 12 patients and on the third day in 6 patients. Nineteen patients underwent two or more bone scans, including all patients with a complicated clinical course.

The mean follow-up was 17 months (4 to 85 months).

## Methods

For bone scintigraphy, 370 MBq 99m-Tc-oxidronate was administered intravenously. Static planar scintigramms of 300 seconds were obtained starting 3 to 4 hours after injection in the anterior and both lateral views. Scans were acquired on a double-head gamma camera (2000XP™, PHILIPS) with a low energy, high resolution collimator in a 128 × 128 matrix. Bone scans were assessed according to a scoring system for tracer uptake ranging from zero to three in comparison to the normal contralateral side (Table 1). Scores of 0 and 1 where considered as ischemic, whereas scores of 2 and 3 as viable.

**Table 1:** Grade Tracer uptake in the graft compared to the contralateral side

0	Absence of tracer uptake
1	Hypofixation/Decreased tracer uptake
2	Normofixation/Same level of tracer uptake
3	Hyperfixation/Increased tracer uptake

We did not perform SPECT investigations because they are more time consuming and, therefore, hardly applicable to patients in the very early postoperative phase.

## Results

Fourty-five patients showed an uncomplicated clinical course with normal early scintigraphic findings (scores 3 or 2). In total, 8 out of 60 grafts were lost (13.3%).

Among the **54 fibular **free flaps, 8 grafts (14.8%) were lost due to necrosis both of the bony part and the skin pedicle.

Seven of these patients (patient 1–7, Table 2) had immediate revision microsurgery. Findings consisted of 6 arterial thrombosis and 1 thrombosis of the vein. The decision for revision surgery was based on ischemia of the skin paddle and poor scintigraphic findings (score 0 in 6 patients and score 1 in 1 patient) in all patients. None of the seven grafts could be saved by revision surgery.

**Table 2:** 

**No.**	**score first bone scan**	score second bone scan (*revision surgery)	**grafted bone**	**clinical course**
**Bone graft lost/poor bone scan findings**				
1.	0	0*	fibula	lost of skin/bone graft
2.	0	1*	fibula	lost of skin/bone graft
3.	0	1*	fibula	lost of skin/bone graft
4.	0	1*	fibula	lost of skin/bone graft
5.	0	*	fibula	lost of skin/bone graft
6.	0	1*	fibula	lost of skin/bone graft
8.	1	*	fibula	lost of skin/bone graft
**Bone graft lost/normal bone scan findings**				
8.	2		fibula	lost of skin/bone graft
**Bone graft lost suspected/local recurrence/poor bone scan findings**				
9.	1	1*	fibula	local recurrence surgery viable graft intraoperatively
**Normal bone scan/thrombosis to skin pedicle/uncomplicated further clinical course**				
10.	2	2*	fibula	uncomplicated
**Poor bone scans/no revision surgery/uncomplicated further clinical course**				
11.	0	3	fibula	uncomplicated
**Poor bone scans/revision surgery/uncomplicated further clinical course**				
12.	1	2*	fibula	uncomplicated
13.	0	2*	scapula	uncomplicated
14.	0	3*	scapula	uncomplicated
15.	1	2*	scapula	uncomplicated

One patient (patient 8, Table 2) showed an uncomplicated course during the first postoperative week with normal scinitigraphic findings. Ten days after surgery, wound healing problems occurred and, subsequently, the skin paddle and bone graft were lost. One patient (patient 9, Table 2) had microvascular revision surgery on the second postoperative day because of ischemia of the skin paddle and a score of 1 in bone scan. In revision surgery, an arterial thrombosis was found and normal vascular patency was established. Whereas the skin paddle showed an uncomplicated clinical course, the bone scan scores remained low (score 1) on two further examinations. Because of local recurrence two months later, a local resection, including fibula graft, had to be performed. Amazingly, a well-vascularized bone graft was found intraoperatively. The defect was reconstructed with a scapular free flap.

One patient (patient 10, Table 2) with fibula free flap had revision surgery because of thrombosis of the vein providing the skin pedicle. Bone scintigraphy was normal (score 2) and the ensuing clinical course was uncomplicated.

One patient (patient 11, Table 2) showed a low score in scintigraphic scans (score 0) but an uncomplicated clinical course. No surgery was performed. A bone scan four days later was normal (score 3) and the ensuing clinical course was uneventful.

**Figure 1:**
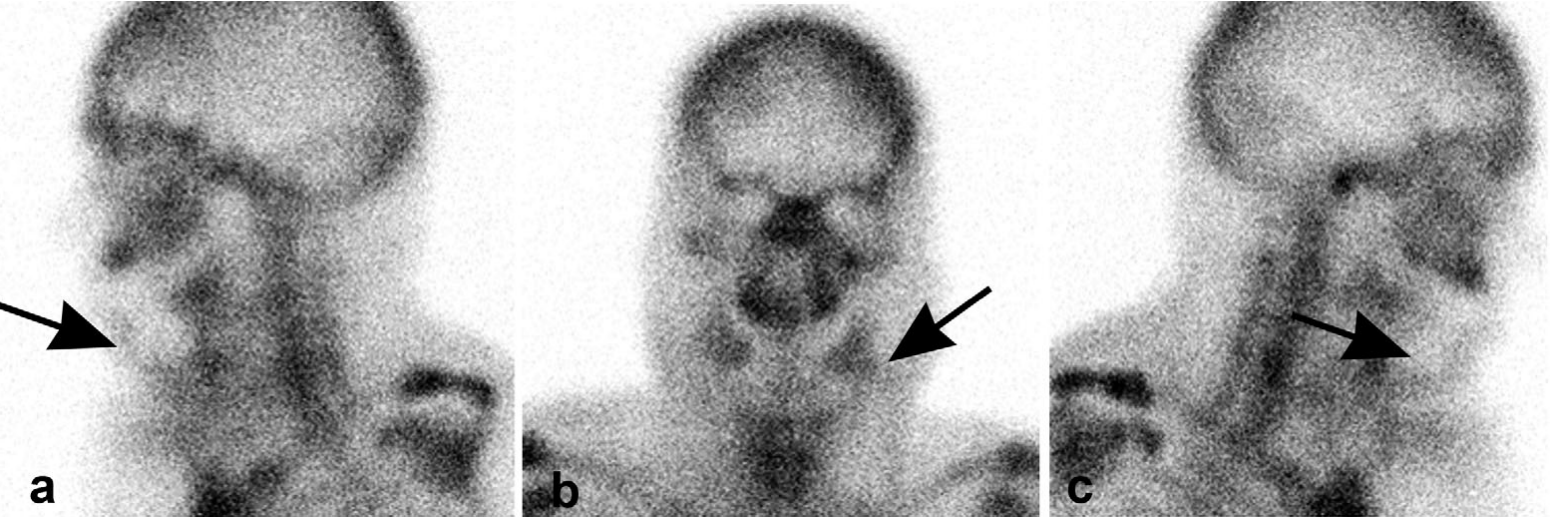
(a: from left, b: anterior, c from right side): Absence of tracer uptake after mandibular reconstruction with fibula free flap on the first postoperative day.

On patient (patient 12, Table 2) had revision surgery because of ischemia of the skin paddle and poor scintigraphic findings (score 1) (figure 1).

**Figure 2:**
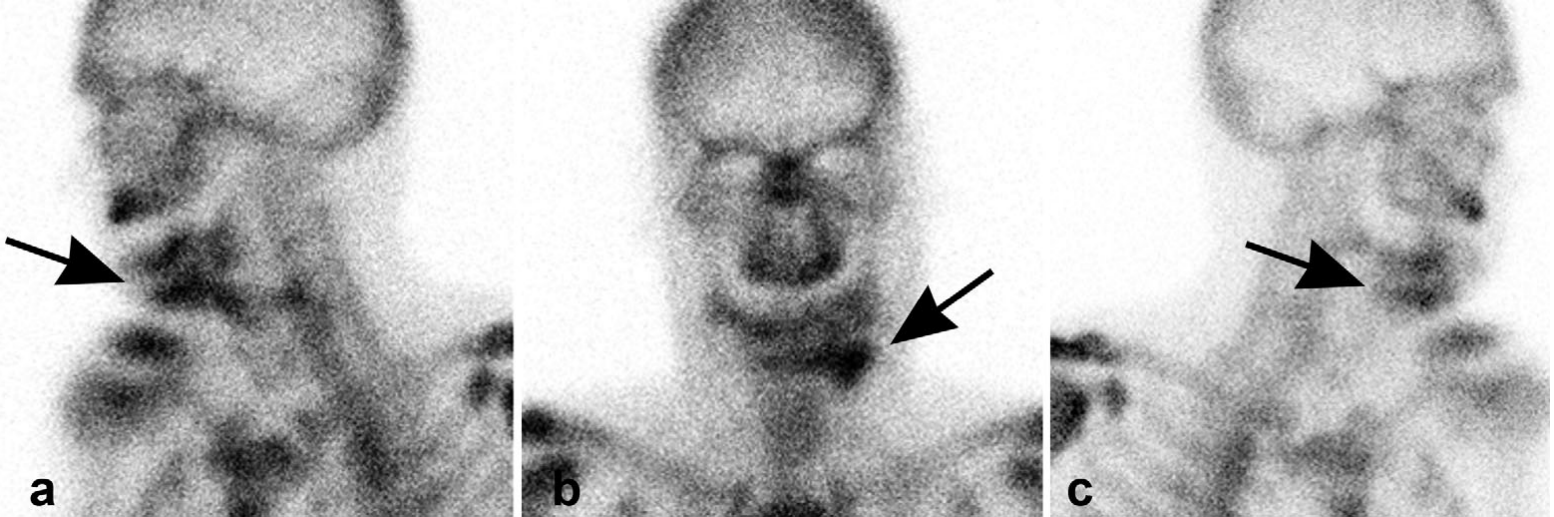
(a: from left, b: anterior, c from right side): Increased tracer uptake of the reconstructed mandible on the third postoperative day after microvascular revision surgery. Vascularisation of the periosteal layer and intramedullary vessels can now be seen.

After microvascular revision surgery, the subsequent clinical course was uncomplicated with normal bone scans (score 2) (figure 2).

None of the **6 scapula **free flaps was lost. Three patients with scapula free flap (patient 13–15, Table 2) had revision microsurgery. One patient had revision surgery because of ischemia of the skin pedicle and poor scintigraphic findings (score 1), whereas two patients had revision surgery because of poor scintigraphic findings only (score 0). Thrombosis was found in all 3 patients. The subsequent clinical course was uncomplicated in all patients, confirmed by normal bone scintigraphic findings (score 2 and 3).

Revision surgery was performed within the first 2 postoperative days in all 13 patients (9 fibula, 3 scapula).

Statistical analysis of early postoperative bone scans showed significantly higher tracer uptake in patients with an uncomplicated clinical course of the bone graft compared to those patients with bone necrosis and/or compromised vascular supply to the bone, found during microvascular revision surgery (p < 10-6, Fisher exact test). For fibula grafts, statistical analysis showed that numbers of osteotomies performed increased the risk for graft failure significantly (p = 0.04, Fisher exact test). We found a tendency to lose grafts in longer grafts and in younger patients (Wilcoxon test). The correlation between scores of the first and the second bone scan was high (r quadrat = 0.45, p = 0.0016, Spearman test) when excluding patients who had had revision surgery.

The **sensivity of early postoperative bone scans **to detect patients with compromised blood supply to the graft was 92% (fibula graft 90%, scapula graft 100%) with a specifity of 98% (fibula graft 97%, scapula graft 100%). The positive predictive value was 92% (fibula graft 90%, scapula graft 100%) and the negative predictive value 97,8% (fibula graft 97%, scapula graft 100%).

The **sensivity of postoperative clinical monitoring**, including direct observation and skin-prick testing to detect patients with a compromised blood supply to the bone graft, was 75% (fibula graft 90%, scapula graft 33%) with a specifity of 98% (fibula graft 97,7%, scapula graft 100%). The positive predictive value was 90% (fibula graft 90%, scapula graft 100%) and the negative predictive value 94% (fibula graft 97%, scapula graft 60%).

## Discussion

As success of reconstructive surgery with microvascular free flaps depends on vascular patency, it is essential to rule out vascular occlusion, either arterial or venous, and monitor flap viability after surgery. Regardless of the experience of the surgeon or the reliability of the donor site, thrombosis is an unavoidable potential complication. Therefore, optimizing microvascular success is based on the ability to identify and salvage failing free flaps immediately. Disa [13] found in his series of 750 free flaps that conventional monitoring techniques, including clinical observation, hand-held Doppler ultrasonography, surface temperature probes and pinprick testing, was highly effective in non-buried free flaps but had not been reliable in buried free flaps. Failing buried free flaps were identified late and found to be unsalvageable on re-exploration. Implantable venous Doppler probes provide "real-time" information regarding both arterial and venous flow and seem to be a promising tool for intraoperative and postoperative monitoring for non-buried and also buried free flaps [8-10]. Several series have described bone scintigraphy as a reliable tool in monitoring microvascular bone grafts, including buried flaps [11,14-19]. Uptake of the radionucleide in the grafted bone is usually interpreted as evidence of bone viability and patent microvascular anastomoses. Metabolically active revascularized bone typically shows normal or diffusely increased tracer uptake. Negative scan results have been significantly associated with later complications [11,14-19] with good sensitivity and specifity in assessing bone graft viability. There is still a debate about the reliability of bone scans performed after the first week postoperatively. Whereas Weiland [20] reported that newly formed bone on the surface of a necrotic graft might lead to false-positive scans, in many others studies [14-16,21] no false positive bone scans on sequential examinations were found. In our studies, the correlation between the first bone scans and later bone scans was high, excluding those patients having had revision surgery. Therefore, it seems reasonable to perform bone imaging once, early after surgery, and immediately, in cases suspicious of vascularized bone graft failure.

However, in all studies to-date, the postoperative bone scans have usually been performed on day 5 to 10 and mostly with regard to long-term complications. In no studies published to date have microvascular reexplorations been performed based on bone scan findings. Our main interest in this study was to discover to which degree bone scans could contribute to early postoperative monitoring and to decide whether or not microvascular revision surgery should be performed. The definite decision to perform microvascular re-explorations was based on clinical and scintigraphic findings.

In a series of 990 consecutive free flaps Kroll [12] found that the majority (80%) of thrombi occurred within the first 2 postoperative days and only few (10%) occurred after the third postoperative day. Based on these studies we performed all bone scans within the first three postoperative days (mean 33 hours postoperatively) and as early as clinical suspicion of complications occurred. In his series, no flaps that developed thrombosis after the third postoperative day were salvaged successfully. He concluded that if flap monitoring had been discontinued after the first 3 postoperative days, their results would have been unchanged.

In several studies, SPECT has been recommended and found superior to planar bone scintigraphy [11,16,17]. Others have found good correlations between SPECT and planar imaging [5,22,23]. We did not perform SPECT investigations because they are more time consuming and are therefore hardly applicable to patients in the very early postoperative phase.

In our series, early bone scans detected a compromised vascular supply to the bone with high accuracy (p < 10-6). The sensitivity of bone scans was superior to the sensitivity of clinical monitoring (92% and 75% respectively). When comparing retrospectively the three monitoring schemes, i.e. clinical monitoring alone, bone scans alone and clinical and bone scan monitoring together, we found the combined monitoring technique to be the most reliable. With clinical monitoring alone, we would have missed 3 patients with a compromised vascular supply to the bone.

If the decision for revision surgery had depended exclusively on bone scans, we would have performed one unnecessary revision surgery, have missed one patient with a compromised vascular supply to the bone and one patient with skin paddle thrombosis, respectively. However most importantly, we were able to salvage two grafts by revision surgery (where thrombosis was found), based exclusively on the bone scan findings. Both patients showed a normal early postoperative clinical course with inconspicuous skin paddles but poor scintigraphic findings. Bone scans were also very useful in buried free flaps where early postoperative monitoring depended exclusively on scans. All patients with buried free flaps showed normal bone scan scores and normal clinical courses.

When bone scans and clinical monitoring were both chosen, one patient with a compromised vascular supply to the bone was overlooked and one patient had unnecessary revision surgery.

Therefore, in our studies, early postoperative scans were a very useful, additional tool in assessing graft viability. Their high sensitivity, which was superior to those of clinical monitoring alone, helped in the decision-making process on whether or not to perform revision surgery. Especially in scapula free flaps, the sensitivity/sensibility (100%/100%) of bone scans to detect compromised vascular supply was excellent and far superior to clinical monitoring alone (33%/100%). All flaps with a compromised vascular supply could be salvaged by microvascular revision surgery.

In contrast in fibula free flaps, the sensitivity/sensibility of bone scans to detect compromised vascular supply was good but, unfortunately, microvascular revision surgery was rarely successful.

During microvascular re-exploration in most cases of fibula grafts, arterial thrombi were found. Because arterial thrombi have been described [12] to occur mostly before the end of the first postoperative day, we might argue that bone scans should be performed even earlier than in our series (mean of 33 postoperative hours).

Whereas increased risk for graft loss in patients with osteotomies and longer bone grafts seems comprehensible, the increased risk (although statistically not significant) for younger patients remains unclear. It might be due to heavy smoking as a risk for both oral cancer and atherosclerosis.
